# Ethylene Modulates Sphingolipid Synthesis in *Arabidopsis*

**DOI:** 10.3389/fpls.2015.01122

**Published:** 2015-12-16

**Authors:** Jian-xin Wu, Jia-li Wu, Jian Yin, Ping Zheng, Nan Yao

**Affiliations:** State Key Laboratory of Biocontrol, Guangdong Provincial Key Laboratory of Plant Resources, School of Life Sciences, Sun Yat-sen UniversityGuangzhou, China

**Keywords:** sphingolipids, ethylene, fumonisin B1, *Arabidopsis*, 1-aminocyclopropane carboxylic acid

## Abstract

Sphingolipids have essential structural and bioactive functions in membranes and in signaling. However, how plants regulate sphingolipid biosynthesis in the response to stress remains unclear. Here, we reveal that the plant hormone ethylene can modulate sphingolipid synthesis. The fungal toxin Fumonisin B1 (FB1) inhibits the activity of ceramide synthases, perturbing sphingolipid homeostasis, and thus inducing cell death. We used FB1 to test the role of ethylene signaling in sphingolipid synthesis in *Arabidopsis thaliana*. The *etr1*-1 and *ein2* mutants, which have disrupted ethylene signaling, exhibited hypersensitivity to FB1; by contrast, the *eto1*-1 and *ctr1*-1 mutants, which have enhanced ethylene signaling, exhibited increased tolerance to FB1. Gene expression analysis showed that during FB1 treatment, transcripts of genes involved in *de novo* sphingolipid biosynthesis were down-regulated in *ctr1*-1 mutants but up-regulated in *ein2* mutants. Strikingly, under normal conditions, *ctr1*-1 mutants contained less ceramides and hydroxyceramides, compared with wild type. After FB1 treatment, *ctr1-*1 and *ein2* mutants showed a significant improvement in sphingolipid contents, except the *ctr1*-1 mutants showed little change in hydroxyceramide levels. Treatment of wild-type seedlings with the ethylene precursor 1-aminocyclopropane carboxylic acid down-regulated genes involved in the sphingolipid *de novo* biosynthesis pathway, thus reducing sphingolipid contents and partially rescuing FB1-induced cell death. Taking these results together, we propose that ethylene modulates sphingolipids by regulating the expression of genes related to the *de novo* biosynthesis of sphingolipids.

## Introduction

Sphingolipids, the complex membrane lipids found ubiquitously in eukaryotes, have important functions in nutrient transport, inflammation, cell differentiation, mitogenesis, migration, apoptosis, senescence, and autophagy (Guenther et al., 2008; Hannun and Obeid, 2008; Hla and Dannenberg, 2012; Sentelle et al., 2012; Maceyka and Spiegel, 2014). A sphingolipid has three parts: a long chain base moiety, a fatty acid moiety, and a head group (Sperling and Heinz, 2003; Pata et al., 2010). According to their molecular structures, plant sphingolipids can be divided into four classes: long-chain bases (LCBs), ceramides, glycosylceramides, and glycosyl inositol phosphoceramides (GIPCs; Pata et al., 2010).

The *de novo* synthesis of sphingolipids starts from a condensation reaction of serine and palmitoyl-CoA by serine palmitoyltransferase (SPT). The resulting 3-keto-dihydrosphingosine is then reduced to a LCB, which forms the backbone of sphingolipids. LCBs can be acylated with various fatty acids, forming dihydroceramides in a reaction catalyzed by ceramide synthases. Ceramides desaturated from dihydroceramides can be modified to complex sphingolipids such as glycosylceramides (GIPCs) or phosphorylated to ceramide phosphates (Hannun and Obeid, 2008; Pata et al., 2010). In the past two decades, research has characterized many of the genes involved in sphingolipid metabolism in plants. SPT, the rate-limiting enzyme of *de novo* sphingolipid synthesis (Tamura et al., 2001), has two subunits (LCB1 and LCB2a, LCB2b), and loss-of-function of either subunit in *Arabidopsis* resulted in lethality (Chen et al., 2006; Dietrich et al., 2008; Teng et al., 2008). LCBs derived from sphinganine (d18:0) can be modified by three enzymes: LCB C-4 hydroxylase, LCB Δ8 desaturase, and LCB Δ4 desaturase (Chen et al., 2008, 2012; Michaelson et al., 2009). LCB kinases phosphorylate LCBs into LCB-1-Ps and four genes (*LCBK1*, *LCBK2*, *SPHK1*, and *SPHK2*) encode these enzymes. LCB-1-Ps participate in abscisic acid signaling (Imai and Nishiura, 2005; Worrall et al., 2008; Dutilleul et al., 2012; Guo et al., 2012). LCB-1-P phosphatases and LCB-1-P lyase dephosphorylate LCB-1-P and affect the dehydration response in *Arabidopsis* (Tsegaye et al., 2007; Nishikawa et al., 2008; Nakagawa et al., 2012). Ceramide synthases encoded by three genes can be divided into two groups based on their substrate preferences: one group includes LOH2, which prefers acyl-CoAs with 16 carbon chain lengths; the other group includes LOH1 and LOH3, which have a wide range of acyl-CoAs as substrates (Markham et al., 2011; Ternes et al., 2011). Loss of the ceramide kinase ACD5 causes the accumulation of ceramides and salicylic acid and impairs plant defenses (Greenberg et al., 2000; Liang et al., 2003; Bi et al., 2014). Inositolphosphorylceramide synthase (IPCS) is involved in RPW8-mediated hypersensitive response-like cell death (Wang et al., 2008). Loss of sphingolipid fatty acid a-hydroxylases results in abnormal plant development and increased sensitivity to oxidative stress (Konig et al., 2012; Nagano et al., 2012). A rice neutral ceramidase prefers ceramides as its substrates (Pata et al., 2008), but the *Arabidopsis* neutral ceramidase *At*NCER1 prefers hydroxyceramides and affects oxidative stress (Li et al., 2015). We also reported that the *Arabidopsis* alkaline ceramidase *At*ACER functions in disease resistance and salt tolerance (Wu et al., 2015). Plants deficient in *AtACER* accumulate ceramides and have reduced levels of LCBs, indicating that *At*ACER is an important regulator of sphingolipid homeostasis (Wu et al., 2015). Although many studies have expanded our understanding of sphingolipids, little is known about how plants regulate sphingolipid metabolism.

Ethylene, a gaseous plant hormone, functions in plant development and stress responses, can be produced by stressed or senescing plants, and affects other plants in the vicinity (Bleecker and Kende, 2000). The ethylene signal transduction pathway has been described in detail (Qiao et al., 2012; Wen et al., 2012; Merchante et al., 2013). Briefly, in the absence of ethylene, the redundant ethylene receptors ETR1, ERS1, ETR2, ERS2, and EIN4 activate the CTR1 protein kinase, which phosphorylates EIN2, inhibiting downstream signaling (Hua and Meyerowitz, 1998; Ju et al., 2012; Qiao et al., 2012; Wen et al., 2012). In the presence of ethylene, the receptors bind ethylene and lose activity, switching off CTR1. Then, the C-terminal of EIN2 is cleaved and transported into the nucleus where it stabilizes the transcription factors EIN3 and EIL1, resulting in the expression of ethylene-responsive genes (Ju et al., 2012; Qiao et al., 2012; Wen et al., 2012).

The fungal toxin Fumonisin B1 (FB1) inhibits the activity of ceramide synthases, resulting in a dramatic increase in the amount of LCBs and ceramides (Abbas et al., 1994; Stone et al., 2000; Markham et al., 2011). Plants treated with FB1 showed hypersensitive response phenotypes, including generation of reactive oxygen species, deposition of phenolic compounds and callose, accumulation of phytoalexins, and expression of pathogenesis-related (PR) genes (Stone et al., 2000). FB1-induced cell death in *Arabidopsis* protoplasts requires jasmonate-, ethylene-, and salicylate-dependent signaling pathways (Asai et al., 2000). Ethylene receptors have distinct roles in FB1-induced cell death in *Arabidopsis*. The *etr1*-1 mutant was more sensitive to FB1 than wild-type plants, but the *ein4*-1 mutant was more tolerant to FB1, and mutants of the other receptor isoforms showed similar responses to FB1 as wild-type plants (Plett et al., 2009). Gene expression analysis revealed that transcripts of *ETR1* and *ETHYLENE RESPONSE FACTOR1* (*ERF1*) increased dramatically in *ein4*-1 mutants after FB1 treatment, indicating that *ETR1* plays a negative role in FB1-induced cell death (Plett et al., 2009). Recently, another group reported that *etr1*-1 mutants were more resistant to FB1, compared with wild-type plants (Mase et al., 2013). The role of ethylene signaling in FB1-induced cell death thus remains to be elucidated.

Previous studies revealed that ethylene regulates the biosynthesis of secondary metabolites such as indole-3-acetic acid, ascorbic acid, flavonoid, and others (Buer et al., 2006; Ruzicka et al., 2007; Gergoff et al., 2010). However, the effects of ethylene on lipid biosynthesis have remained unclear. Here, we report that ethylene modulates sphingolipid synthesis. Our results showed that ethylene signaling plays a negative role in FB1-induced cell death and down-regulates the expression of genes involved in *de novo* sphingolipid biosynthesis, thereby reducing sphingolipid synthesis.

## Materials and Methods

### Plant Materials and Growth Conditions

The *etr1*-1 (CS237), *ein2* (CS8844), *ctr1*-1(CS8057), *eto1*-1 (CS3072), and *acer*-1 (CS876510, Wu et al., 2015) mutants were obtained from the *Arabidopsis* Biological Resource Center (ABRC); the double mutant *ctr1 ein2* was a gift from Dr. Chi-Kuang Wen. All the mutants used in this study were in the *Arabidopsis thaliana* ecotype Columbia (Col-0), which was used as the wild-type control. FB1, 1-aminocyclopropane-1-carboxylic acid (ACC), and 3,3-diaminobenzidine-HCl (DAB) were purchased from Sigma.

Seeds were sterilized and plated on 1/2x MS solid medium (1% sucrose, 0.8% agar), stratified in the dark for 2 days at 4°C, and then transferred into an incubator under a 16 h light/8 h dark cycle (4000 lux light intensity) at 22°C.

### Chemical Treatments

For germination assays, 1/2x MS solid medium was supplemented with various combinations of 0.5 μM FB1 and 50 μM ACC. For sphingolipid analysis, seeds were germinated on 1/2x MS solid medium for 7 days, and the resulting seedlings were transferred to 1/2x MS solid medium supplemented with various combinations of 0.5 μM FB1 and 50 μM ACC for another 6 or 8 days under the same conditions. For gene expression, 7-day-old seedlings were transferred to 1/2x MS solid medium supplemented with 0.5 μM FB1 or 50 μM ACC, and harvested at 24 or 48 h.

### DAB Staining

For analysis of the FB1-induced oxidative burst, 7-day-old seedlings were transferred to 1/2x MS solid medium supplemented with 0.5 μM FB1, harvested at 0, 12, 24, and 48 h. The seedlings were quickly immersed in 1 mg/ml DAB, then incubated for 3 h in the dark. The pigments in the DAB-stained seedlings were removed with acetic acid/glycerol/ethanol (1:1:3). DAB staining was observed under a stereomicroscope (SteREO Lumar.V12 Carl Zeiss) equipped with a CCD camera (AxioCam MRc, Carl Zeiss) and quantified by measuring the intensity per unit area, with Image J. At least six leaves per genotype were measured at each time point and three independent experiments were performed.

### Sphingolipid Analysis

Sphingolipids were extracted as previously described (Wu et al., 2015). Briefly, about 100 mg of fresh seedlings was homogenized. Sphingolipids were extracted in the lower phase of isopropanol/hexane/water (v/v/v, 55:20:25) at 60°C for 15 min. After centrifugation, the supernatants were air-dried, de-esterified in 33% methylamine in ethanol/water (7:3, v/v), air-dried and re-dissolved in 1 ml methanol, and then analyzed with a Shimadzu UFLC XR/TripleTOF 5600 LC/MS system using an Agilent Eclipse XDB C8 column (50 mm × 2.1 mm, 1.8 μm). Quantification was performed based on peak area and internal standards. C17 base D-erythro-sphingosine and d18:1 C12:0-ceramide were used as internal standards.

### Quantitative RT-PCR Analysis

Total RNA was extracted using the E.Z.N.A. Plant RNA Kit (Cat#R6827-01, OMEGA). The first-strand cDNA was synthesized from 1 μg of total RNA using the PrimeScript RT reagent Kit (TAKARA, DRR047A). Real-time PCR was performed using the SYBR Premix Ex Taq kit (Takara, RR820L) according to the manufacturer’s instructions, and quantitatively analyzed by Step One Plus Real-Time PCR Systems (AB SCIEX). Relative expression of genes was determined by applying the 2^-ΔΔ^CT method and using *ACT2* for normalization. The primers for amplification are listed in **Supplementary Table S1**.

## Results

### Enhanced Ethylene Signaling Rescues FB1-Induced Cell Death

**FIGURE 1:**
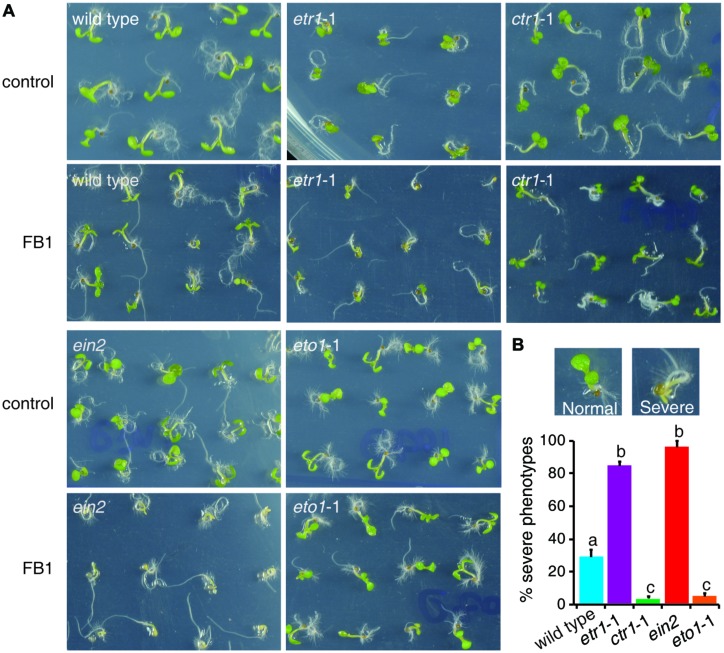
**Responses of mutants involved in ethylene signaling to FB1 treatment.** **(A)** The wild-type, *etr1*-1, *ein2*, *ctr1*-1, and *eto1*-1 seeds were germinated on 1/2x MS with or without 0.5 μM FB1. Photos were taken after 6 days of treatment. **(B)** Quantitation of the phenotypes in **(A)**. Seedlings with cotyledon bleaching were scored as having a severe phenotype. The data represent means of three independent experiments. At least 40 seeds of each sample were used in each experiment. Data are means ± SD (*n* = 3) and sets marked with different letters indicate significances assessed by Student–Newman–Keul test (*P* < 0.05).

The fungal toxin FB1, a well-known ceramide synthase inhibitor, provides a useful tool to study how plants regulate sphingolipid synthesis. As ethylene plays a pivotal role in FB1-induced cell death (Plett et al., 2009), we inferred that ethylene signaling may affect sphingolipid synthesis. To address this hypothesis, we selected mutants that affect ethylene signaling. The dominant negative *ethylene receptor1* (*etr1*) mutant *etr1*-1 causes loss of ethylene perception (Bleecker et al., 1988). The *constitutive triple response1* (*ctr1*) mutant *ctr1*-1 causes the loss of CTR1 kinase activity, and thus shows continuous activation of ethylene signaling (Kieber et al., 1993). The *ethylene insensitive2* (*ein2*) mutant is impaired in ethylene signaling (Alonso et al., 1999). The *ethylene-overproducing1* (*eto1*) mutant *eto1*-1, which produces excess ethylene, exhibits a constitutive ethylene response (Wang et al., 2004). Seeds of wild type, *etr1*-1, *ctr1*-1, *ein2*, and *eto1*-1 were germinated on half-strength Murashige and Skoog medium (1/2x MS) with or without 0.5 μM FB1. Phenotypes were sorted and analyzed after 6 days of treatment. Interestingly, about 30% of wild-type seedlings showed a severe phenotype, but only about 5% of *ctr1*-1 and *eto1*-1 seedlings showed severe phenotypes, and more than 85% of *ert1*-1, and *ein2* seedlings showed severe phenotypes (**Figure 1**). Thus, the constitutive ethylene response mutants (*ctr1*-1 and *eto1*-1) exhibited more resistance to FB1 and the mutants deficient in ethylene signaling (*etr1*-1 and *ein2*) displayed more sensitivity to FB1.

**FIGURE 2:**
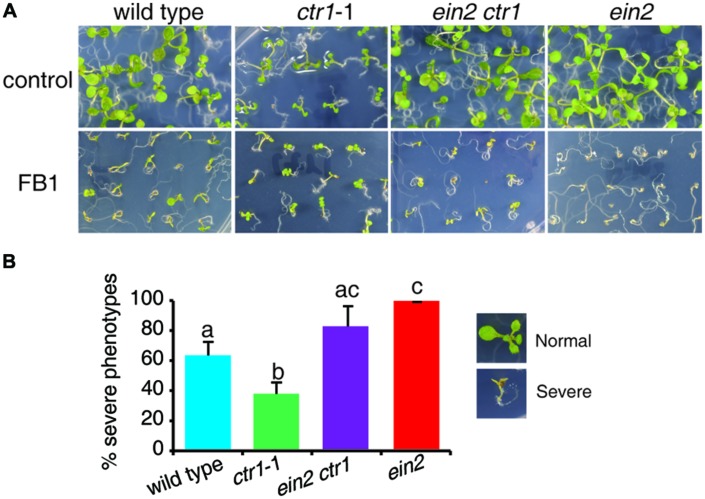
**Phenotypes of FB1-treated wild-type, *ctr1*-1, *ein2 ctr1*-1, and *ein2* plants.** **(A)** The wild-type, *ctr1*-1, *ein2 ctr1*-1, and *ein2* seeds were germinated on 1/2x MS with or without 0.5 μM FB1. Photos were taken after 8 days of treatment. **(B)** Quantitation of the phenotypes in **(A)**. Seedlings with cotyledon bleaching were scored as having a severe phenotype. The data represent means of three independent experiments. At least 40 seeds of each sample were used in each experiment. Data are means ± SD (*n* = 3) and sets marked with different letters indicate significances assessed by Student–Newman–Keul test (*P* < 0.05).

Recently, a genetic screen for suppressors of *ctr1*-1 identified a *ctr1 ein2* double mutant, which showed no response to ethylene (Zhang et al., 2014). To further confirm the role of ethylene signaling in FB1-induced cell death, we tested the responses of the *ctr1 ein2* mutant to FB1 treatment. The *ctr1 ein2* double mutant was hypersensitive to FB1, similar to the *ein2* single mutants (**Figure 2**), indicating that ethylene signaling plays a vital role in the response to FB1.

**FIGURE 3:**
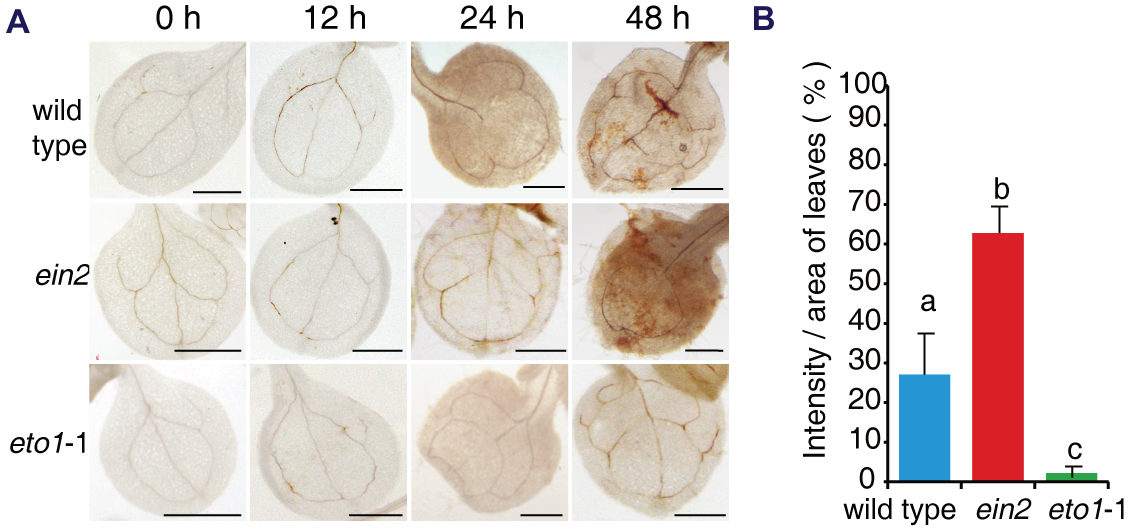
**Histochemical detection of H_2_O_2_ in leaves of mutants after treatment with FB1.** **(A)** Representative leaves of wild-type, *ein2*, and *eto1*-1 seedlings stained with DAB after treatment with 0.5 μM FB1 for the indicated times. **(B)** Quantification of DAB staining in leaves treated with FB1 for 48 h. H_2_O_2_ deposits were quantified by measuring the intensity of DAB staining per unit area of leaves, using Image J. At least six leaves per genotype were analyzed for each time point and the experiments were repeated three times with similar results. Values represent means ± SD (*n* = 6). Data sets marked with different letters indicate significant differences assessed by Student–Newman–Keul test (*P* < 0.05).

Previous studies have shown that FB1 induces an oxidative burst, which may result in cell death (Stone et al., 2000; Shi et al., 2007). We asked whether the effects of ethylene signaling mutants on FB1 affected the oxidative burst. Seven-day-old seedlings of wild type, *ein2*, and *eto1*-1 were transferred to 1/2x MS supplemented with 0.5 μM FB1, and then seedlings were harvested at the indicated times. H_2_O_2_ was visualized by staining with diaminobenzidine (DAB). When seedlings were treated with FB1, we observed a significant increase in DAB staining in *ein2* leaves, but a significant decrease in DAB staining in *eto1*-1 leaves compared to wild-type plants (**Figure 3**). These results indicate that ethylene signaling plays an important role in FB1-induced cell death.

### Ethylene Signaling in Ceramidase Mutants Negatively Affects FB1-Induced Cell Death

**FIGURE 4:**
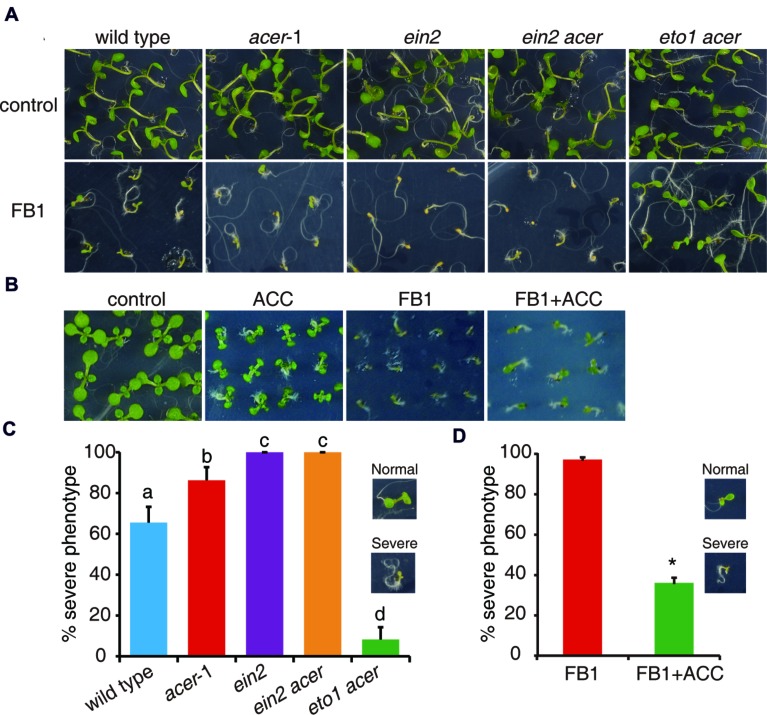
**Phenotypes of FB1-treated wild-type, *acer-1, ein2, ein2 acer*, *and eto1 acer* plants.** **(A)** Seeds were germinated on 1/2x MS with or without 0.5 μM FB1. Photos were taken after 8 days of treatment. **(B)** ACC reduces FB1-induced cell death in *acer*-1 mutant. Seeds were germinated on 1/2x MS supplemented with various combinations of 0.5 μM FB1 and 50 μM ACC. Photos were taken after 2 weeks of treatment. **(C)** Quantitative analysis of the phenotype of seedlings in **(A)**. Data presented are means ± SD (*n* = 3) and sets marked with different letters indicate significances assessed by Student–Newman–Keul test (*P* < 0.05). **(D)** Quantitative analysis of the phenotype of seedlings in **(B)**. Data presented are means ± SD (*n* = 3) and asterisk indicates *P* < 0.01 using Student’s *t*-test.

To further test the idea that enhanced ethylene signaling could affect FB1-induced cell death, we employed a genetic approach using the mutants involved in sphingolipid synthesis. *AtACER* encodes an alkaline ceramidase that plays a critical role in sphingolipid homeostasis in *Arabidopsis* (Wu et al., 2015). *acer*-1, a T-DNA insertion mutant of *AtACER*, was hypersensitive to FB1 compared to wild type (Wu et al., 2015). By crossing *ein2* and *eto1* with *acer*, we generated *ein2 acer* and *eto1 acer* double mutants. As shown in **Figure 4**, we found that *ein2 acer* double mutant plants were more sensitive to FB1 than *acer*-1 mutant plants, but *eto1 acer* double mutants showed significantly increased resistance to FB1 (**Figures 4A,C**). The data indicated that blocking ethylene signaling enhanced the toxicity of FB1 and strengthening ethylene signaling decreased the toxicity of FB1. Interestingly, ACC significantly enhanced the tolerance of *acer*-1 mutants to FB1 (**Figures 4B,D**). Taken together, our results indicate that ethylene signaling in *acer*-1 mutants plays a negative role in FB1-induced cell death.

### Ethylene Signaling Inhibits Sphingolipid Synthesis

**FIGURE 5:**
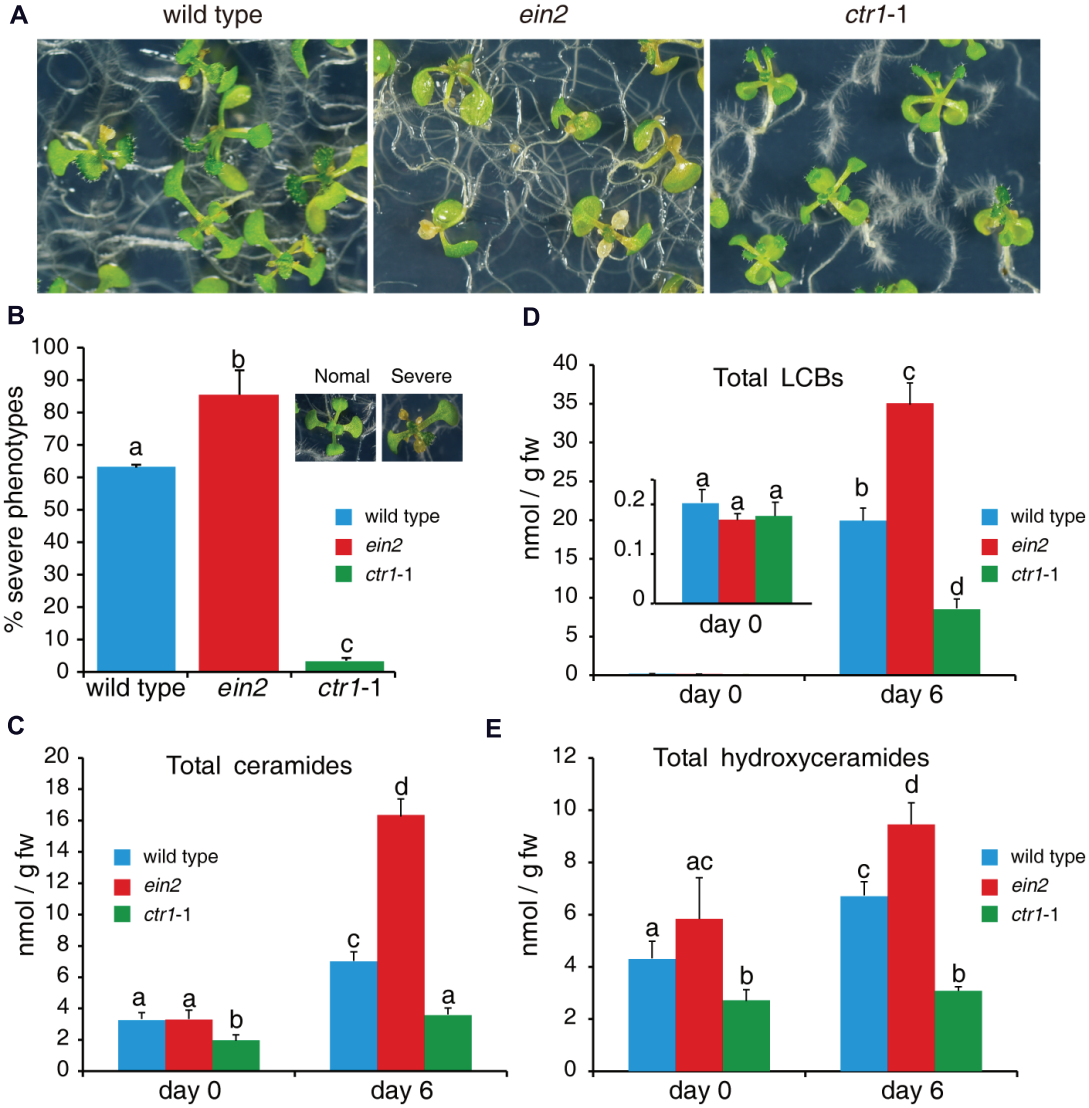
**Sphingolipid profiles of wild-type, *ein2*, and *ctr1*-1 seedlings during FB1 treatment.** Seven-day-old seedlings were treated with 0.5 μM FB1 and collected after 6 days. Total sphingolipids were extracted and analyzed by LC–MS system. **(A)** Phenotypes of FB1-treated seedlings. Photos were taken after 6 days of treatment. **(B)** Quantitation of the phenotypes in **(A)**. Seedlings with lesions were scored as having a severe phenotype. The data represent at least three independent experiments. More than 100 seedlings were counted. **(C–E)** Comparisons of different kinds of sphingolipids in wild-type, *ein2*, and *ctr1*-1 seedlings. Data represent means ± SD from three independent biological experiments and sets marked with different letters indicate significances assessed by Student–Newman–Keul test (*P* < 0.05). **(C)** LCBs. **(D)** Ceramides. **(E)** Hydroxyceramides. See **Supplementary Figure S1** for the detailed analysis.

Fumonisin B1 inhibits synthesis of very-long-acyl-chain (C > 18 carbons) ceramides and activates SPT, resulting in accumulation of LCBs and long-acyl-chain ceramides (C16; Abbas et al., 1994; Shi et al., 2007; Markham et al., 2011). FB1-induced cell death may occur through priming of downstream signaling by LCBs and ceramides (Shi et al., 2007; Wu et al., 2015). As we found that mutants of ethylene signaling pathways showed different phenotypes in response to FB1 treatment (**Figures 5A,B**), we analyzed the profiles of sphingolipids in wild type, *ctr1*-1, and *ein2* with or without FB1 treatment. No significant difference was detected in the contents of LCBs among the mutants without FB1 treatment (**Figure 5C**); however, the *ctr1*-1 mutants had less ceramides and hydroxyceramides than the wild-type and *ein2* plants (**Figures 5D,E**). After 6 days of FB1 treatment, the amounts of LCBs (especially t18:0 and d18:0) dramatically increased in all of the mutants, although *ein2* showed a larger change and *ctr1*-1 showed a smaller change compared to wild type (**Figure 5C**). Total ceramides increased more than fivefold in *ein2* mutants, but increased only twofold in wild type and 1.7-fold in *ctr1*-1 mutants, compared with untreated plants (**Figure 5D**).

We also compared ceramide species with different length fatty acid moieties, LCB moieties, or saturated or unsaturated fatty acid moieties (**Supplementary Figure S1**). Again, the data showed that the *ctr1*-1 mutants had less ceramide accumulation after FB1 treatment (**Supplementary Figures S1A**–**C**). The *ctr1*-1 mutants showed no significant change in total hydroxyceramides after FB1 treatment, whereas the *ein2* mutants and wild type showed slight increases (**Figure 5E**). Overall, under normal growth conditions, the *ctr1*-1 mutants had less ceramides and hydroxyceramides compared to wild type and *ein2* mutants. However, when seedlings were treated with FB1, the LCB, ceramide, and hydroxyceramide contents increased in wild type and *ein2* especially; by contrast, only LCBs and ceramides accumulated in *ctr1*-1 mutants, and their contents were lower than in wild type, implying that ethylene signaling may negatively regulate sphingolipid synthesis.

**FIGURE 6:**
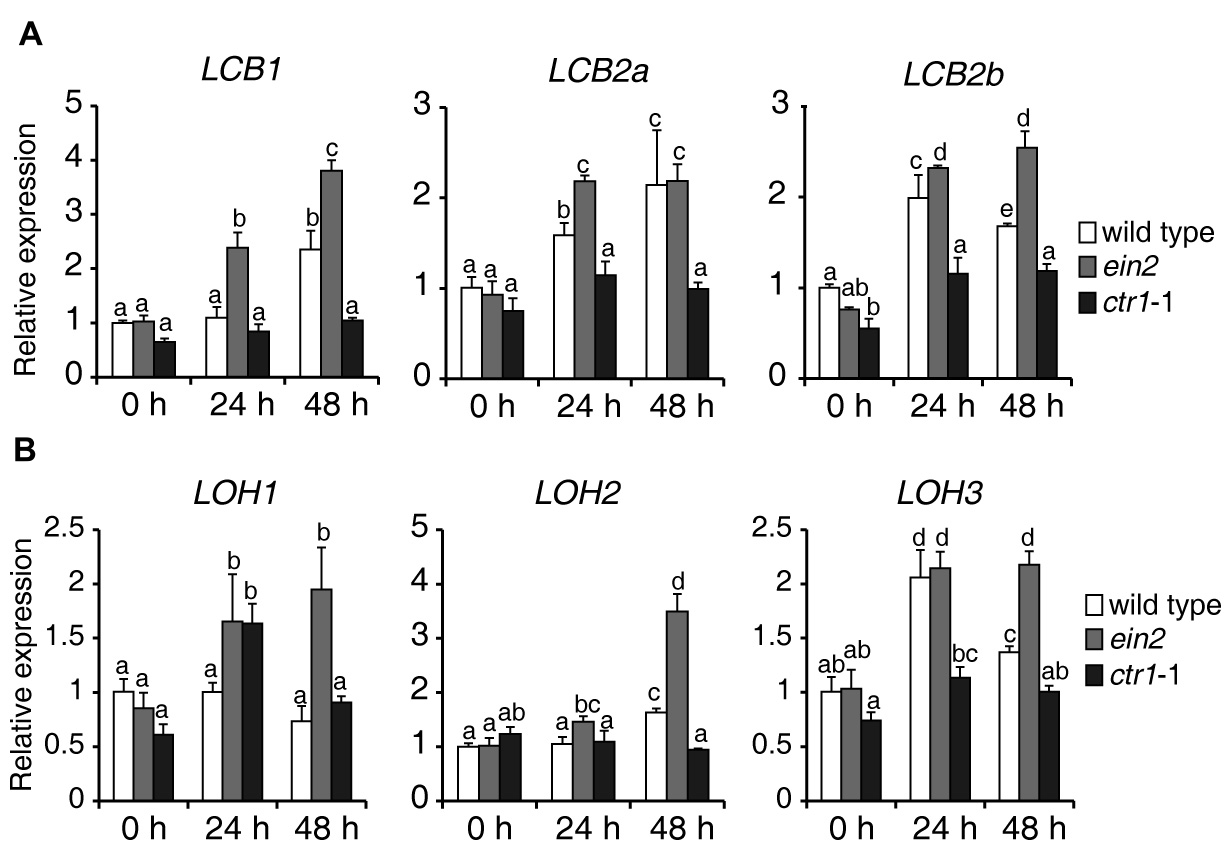
**Expression of genes involved in *de novo* sphingolipid synthesis in wild-type, *ein2*, and *ctr1*-1 seedlings during FB1 treatment.** Seven-day-old seedlings were treated with 0.5 μM FB1 and samples were collected at the indicated times. *ACT2* (AT3G18780) was used as an internal control. Gene expression values are presented relative to average wild-type levels, which were set as 1. Data represent means ± SD from three technical repeats and sets marked with different letters indicate significances assessed by Student–Newman–Keul test (*P* < 0.05). This experiment was repeated three times with similar results. The primers used for this analysis are provided in **Supplementary Table S1**. **(A)** Relative transcript abundance of genes involved in LCB synthesis (*LCB1*, *LCB2a*, *LCB2b*). **(B)** Relative transcript abundance of genes involved in ceramide synthesis (*LOH1*, *LOH2*, *LOH3*).

To explore how ethylene signaling regulates sphingolipid synthesis, 7-day-old seedlings of wild type, *ein2*, and *ctr1*-1 mutants were transferred to 1/2x MS supplemented with 0.5 μM FB1, harvested at the indicated times, and used for measurement of expression of genes involved in *de novo* sphingolipid synthesis. Surprisingly, during FB1 treatment, almost all of the analyzed genes showed higher expression in *ein2* mutants, but lower expression in *ctr1*-1 mutants, compared to wild type (**Figure 6**), suggesting that ethylene signaling modulates sphingolipids, possibly by regulating the expression of genes that participate in *de novo* synthesis of sphingolipids.

### ACC Inhibits Sphingolipid Synthesis

**FIGURE 7:**
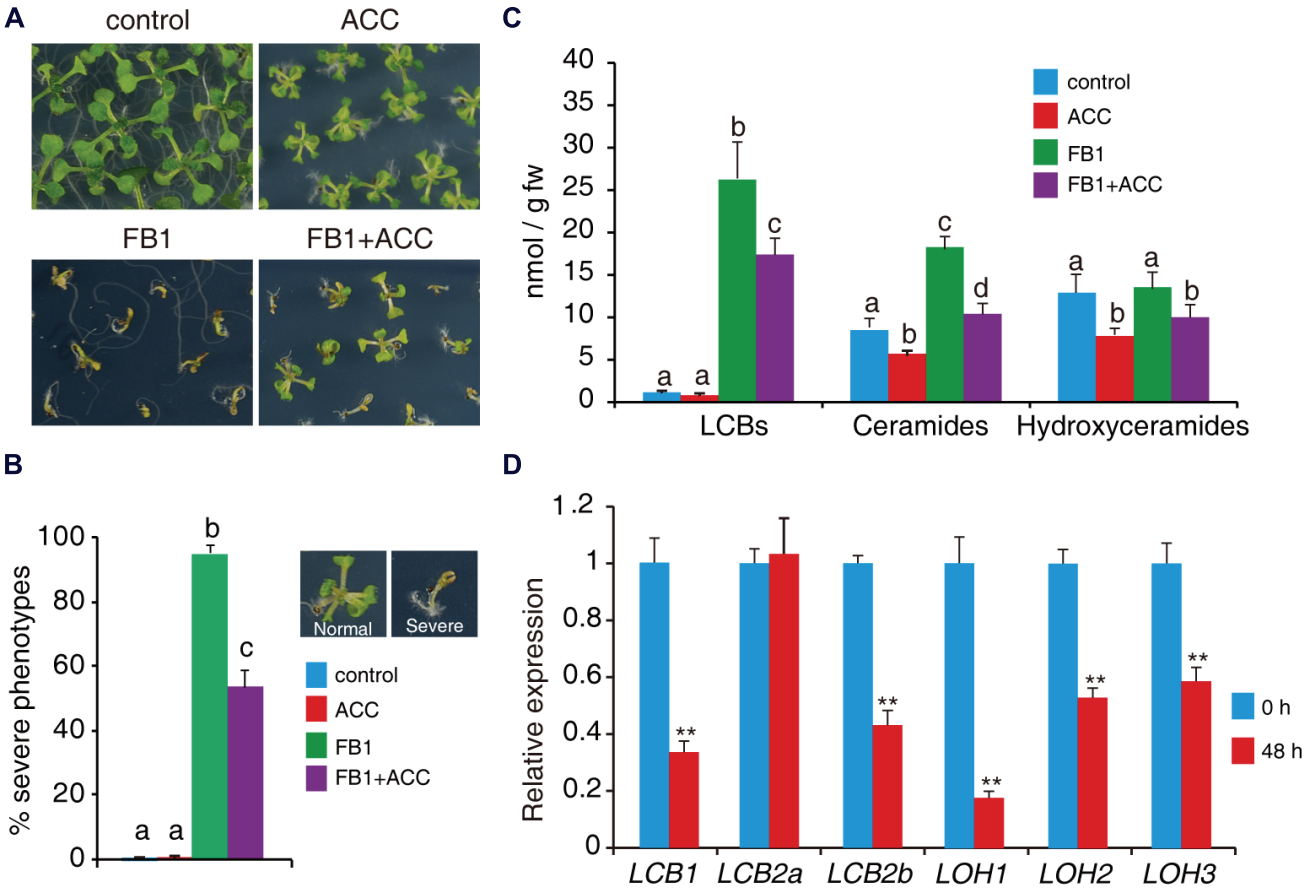
**ACC reduces FB1-induced cell death.** **(A)** Wild-type seeds were germinated on 1/2x MS supplemented with various combinations of 0.5 μM FB1 and 50 μM ACC. Photos were taken after 2 weeks of treatment. **(B)** Quantitative analysis of the phenotype of seedlings in **(A)**. At least 100 seeds of each sample were used in each experiment. Data presented are means ± SD (*n* = 3) and sets marked with different letters indicate significances assessed by Student–Newman–Keul test (*P* < 0.05). **(C)** Sphingolipid analysis of wild-type seedlings treated with various combinations of ACC and FB1. One-week-old seedlings were used and harvested after 6 days of treatment. Total sphingolipids were extracted and analyzed (see **Supplementary Figure S2** for the detailed analysis). **(D)** Expression of genes involved in *de novo* sphingolipid synthesis in wild-type seedlings after 48 h ACC treatment. Data represent means ± SD from three technical repeats and sets marked with double asterisks indicate significance assessed by Student’s *t*-test (*P* < 0.01). This experiment was repeated three times with similar results.

To further assess the role of ethylene in sphingolipid synthesis, we tested the effects of the ethylene biosynthesis precursor ACC on FB1-induced cell death. Wild-type seeds were germinated on 1/2x MS supplemented with combinations of 0.5 μM FB1 and 50 μM ACC. As shown in **Figure 6A**, wild-type plants treated with ACC showed a relatively small stature, similar to *ctr1*-1 mutants. We found that ACC clearly reduced FB1-induced cell death (**Figures 7A,B**). To investigate whether ethylene affects synthesis of sphingolipids, 1-week-old seedlings of wild type were treated with combinations of 0.5 μM FB1 and 50 μM ACC for 6 days, and then sphingolipids were extracted and analyzed. Interestingly, ACC and *ctr1*-1 showed similar effects on sphingolipid contents. The amounts of ceramides and hydroxyceramides decreased after ACC treatment, compared to control (**Figure 7C**). Seedlings treated with FB1 plus ACC showed dramatically decreased sphingolipid accumulation including LCBs, hydroxyceramides, and ceramides (**Figure 7C**), indicating that ACC may antagonize FB1-induced sphingolipid synthesis. We next analyzed gene expression after ACC treatment. The genes involved in *de novo* sphingolipid synthesis were almost all down-regulated (**Figure 7D**), implying that ACC-mediated inhibition of synthesis of sphingolipids may be achieved by regulating expression of these genes.

## Discussion

Sphingolipids have emerged as a bioactive lipids involved in many aspects of plant growth and responses to biotic or abiotic stresses. However, how plants regulate sphingolipid biosynthesis, and the relationship between sphingolipids and ethylene, remain unclear. In this study, we explored the role of ethylene signaling in sphingolipid synthesis by using the ceramide synthase inhibitor FB1. We demonstrated that ethylene can modulate sphingolipids by regulating the expression of genes related to the *de novo* biosynthesis of sphingolipids.

Under our experimental conditions, the *etr1*-1 mutant exhibited enhanced susceptibility to FB1 compared to wild-type plants, similar to the results reported by Plett et al. (2009). However, the plants used in other studies were cultivated under different photoperiod conditions, and these studies produced different results (Mase et al., 2013). The conflicting observations on the roles of ETR1 in FB1-induced cell death may be because plants use combinations of ethylene receptors to respond to various environments (Liu and Wen, 2012; Wilson et al., 2014). CTR1 and its downstream target EIN2 function as core components in the ethylene signaling pathway (Qiao et al., 2012; Wen et al., 2012). The roles of *CTR1* and *EIN2* in FB1-induced cell death remain critical questions. We found that the *ctr1*-1 mutant was more tolerant to FB1 compared to wild-type plants, whereas the *ein2* mutant was much more susceptible to FB1. In addition, the *ctr1 ein2* double mutant showed hypersensitivity to FB1, as did *ein2*, but not *ctr1*-1 mutants. Also, exogenous ACC could partially rescue the phenotype of FB1-treated seedlings. These data indicated that ethylene signaling plays a negative role in FB1-induced cell death. Consistent with previous reports, plants treated with FB1 resulted in a dramatic accumulation of LCBs and long chain ceramides (Abbas et al., 1994; Shi et al., 2007; Markham et al., 2011). The ceramidase mutant *acer*-1 with a higher ceramide levels, was more sensitive to FB1 compared to wild-type plants (Wu et al., 2015). Interestingly, the *ein2 acer* double mutant showed enhanced sensitivity to FB1, but the *eto1 acer* mutant showed reduced sensitivity to FB1. Moreover, FB1-treated *acer*-1 seedlings could be partially rescued by exogenous ACC. As LCBs and ceramides both can induce PCD in plants, we suggest that ethylene signaling may regulate FB1-induced cell death by modulating sphingolipid synthesis.

Although our understanding of sphingolipids has improved, little is known about how plants regulate sphingolipid metabolism. In yeast and human cells, Orm proteins control *de novo* synthesis of sphingolipids by inhibiting the activity of SPT, which participates in a phosphorylation-based feedback loop (Breslow et al., 2010; Roelants et al., 2011). In plants, however, we still lack evidence showing that certain proteins regulate sphingolipid synthesis. In this work, to test the role of ethylene in sphingolipid synthesis, we employed mutants affecting the ethylene signaling pathway and investigated their effects on FB1-induced perturbation of sphingolipids. The analysis of sphingolipids showed that before FB1 treatment, the amounts of LCBs did not significantly differ in wild-type, *ctr1*-1, and *ein2* seedlings, but the *ctr1*-1 mutants had less ceramides and hydroxyceramides. When seedlings were treated with FB1, LCBs, ceramides and hydroxyceramides accumulated in wild-type and *ein2* seedlings, with more accumulation in the *ein2* seedlings; by contrast, we detected only limited accumulation of LCBs and ceramides in *ctr1*-1 seedlings. These results indicated that ethylene signaling may negatively regulate sphingolipids. To further explore the effects of ethylene on sphingolipid synthesis, we used ACC to mimic the action of ethylene. We found that ACC treatment resulted in lower ceramide and hydroxyceramide contents in seedlings compared to control, and could restrict FB1-induced sphingolipid synthesis. Gene expression analysis showed that almost all of the genes related to *de novo* sphingolipid synthesis were up-regulated in *ein2* mutants but down-regulated in *ctr1*-1 mutants after FB1 treatment, and these genes were down-regulated after ACC treatment. Taken together, these data suggested that ethylene may modulate *de novo* synthesis of sphingolipids. As transcription factors are vital to the regulation of lipid synthesis (Kannangara et al., 2007; Raffaele et al., 2008), we also speculate that ethylene-responsive transcription factors may regulate the expression of genes that participate in sphingolipid synthesis.

In the past two decades, flourishing studies on sphingolipids in plants have revealed that sphingolipids have critical functions in plant morphogenesis, development, senescence, and resistance to biotic and abiotic stress. Ethylene pervades every aspect of plant life. Our findings illustrate the important connections between these two bioactive molecules and indicate that ethylene may modulate *de novo* synthesis of sphingolipids. Future studies will focus on the molecular mechanism by which ethylene modulates the expression of genes involved in sphingolipid synthesis.

## Conflict of Interest Statement

The authors declare that the research was conducted in the absence of any commercial or financial relationships that could be construed as a potential conflict of interest.
