# Supplementary material for: Ethylene Modulates Sphingolipid Synthesis in Arabidopsis
Source: Front Plant Sci. 2015 Dec 16;6:1122. doi: 10.3389/fpls.2015.01122 (PMC4679861; doi:10.3389/fpls.2015.01122)
Supplement: Supplementary file 1 [file Table_1.DOCX]

**Supplemental Table S1. Primers used in this study**

| Gene loci | Name | Sequence |
| --- | --- | --- |
| AT4G36480 | LCB1-F | CTTCTTAAAGCGTCGGAGTCA |
|  | LCB1-R | TCTGCGGATTCTGTTGTCTAC |
| AT5G23670 | LCB2a-F | TGGCTATATTGCTGGATCTAAGGA |
|  | LCB2a-R | GTGCGGAAGGAGTTGGTATG |
| AT3G48780 | LCB2b-F | CAGGTGTTATCGCAGTCATCTT |
|  | LCB2b-R | GCACATTCGTGGTCTGGAAG |
| AT3G25540 | LOH1-F | TCCGATTCTGAAAGCGATGATG |
|  | LOH1-R | ATTCCTAGTCTCCGTGTGGTT |
| AT3G19260 | LOH2-F | GGATTCTTCTTCTTGAGGCTTGTC |
|  | LOH2-R | CCGAGTAGCAGCATCATTCAAT |
| AT1G13580 | LOH3-F | CTCTCCTATATTGCTTGCTTGTTCT |
|  | LOH3-R | AATCAGTCTTCGTGCTCATCTTC |
| AT3G18780 | ACT2-F | GGTAACATTGTGCTCAGTGGTGG |
|  | ACT2-R | GGTGCAACGACCTTAATCTTCAT |
