# Supplementary figures and images for: Ethylene Modulates Sphingolipid Synthesis in Arabidopsis
Source: Front Plant Sci. 2015 Dec 16;6:1122. doi: 10.3389/fpls.2015.01122 (PMC4679861; doi:10.3389/fpls.2015.01122)

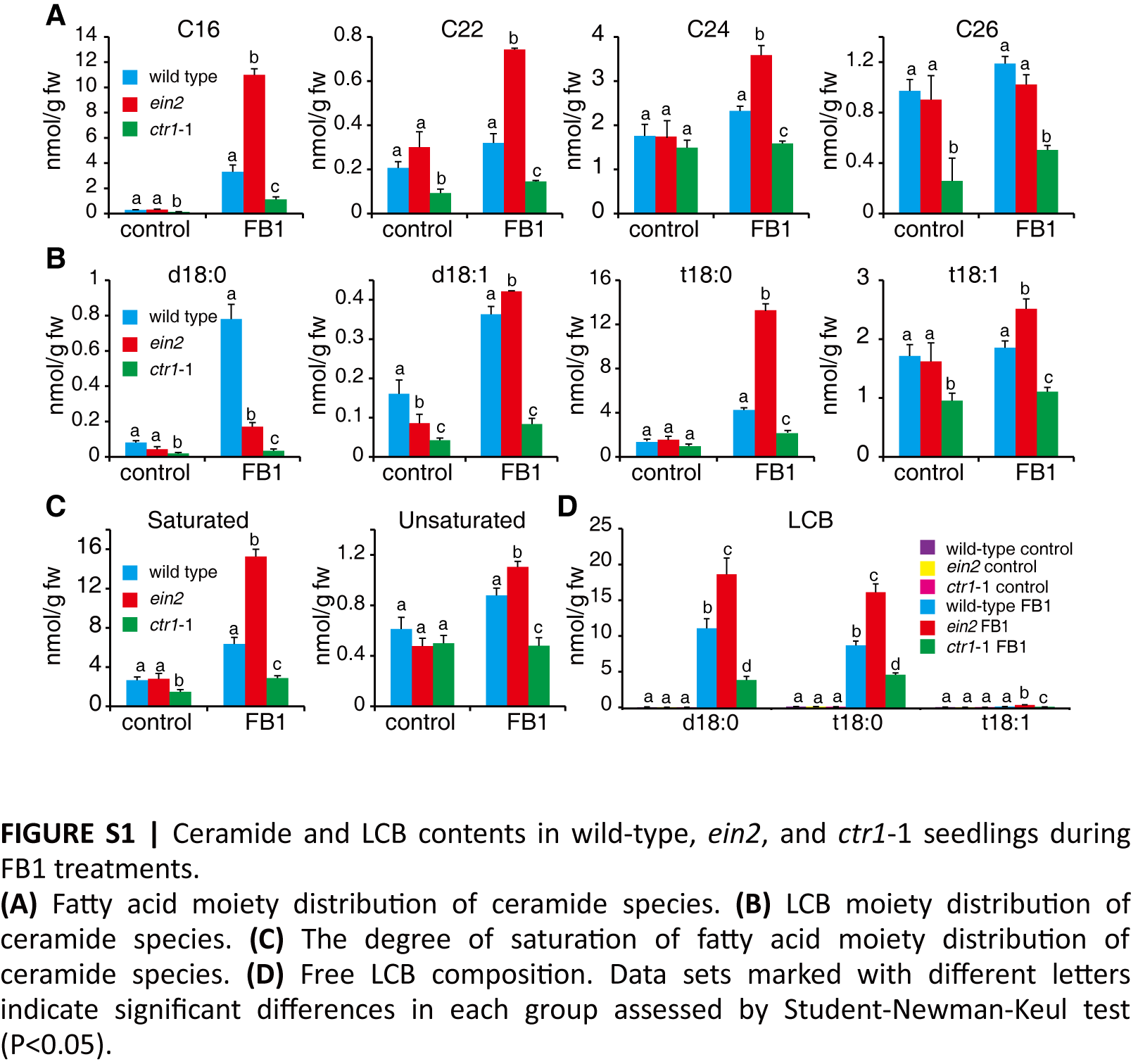

Supplement: Supplementary file 2 [file Image_1.TIF]

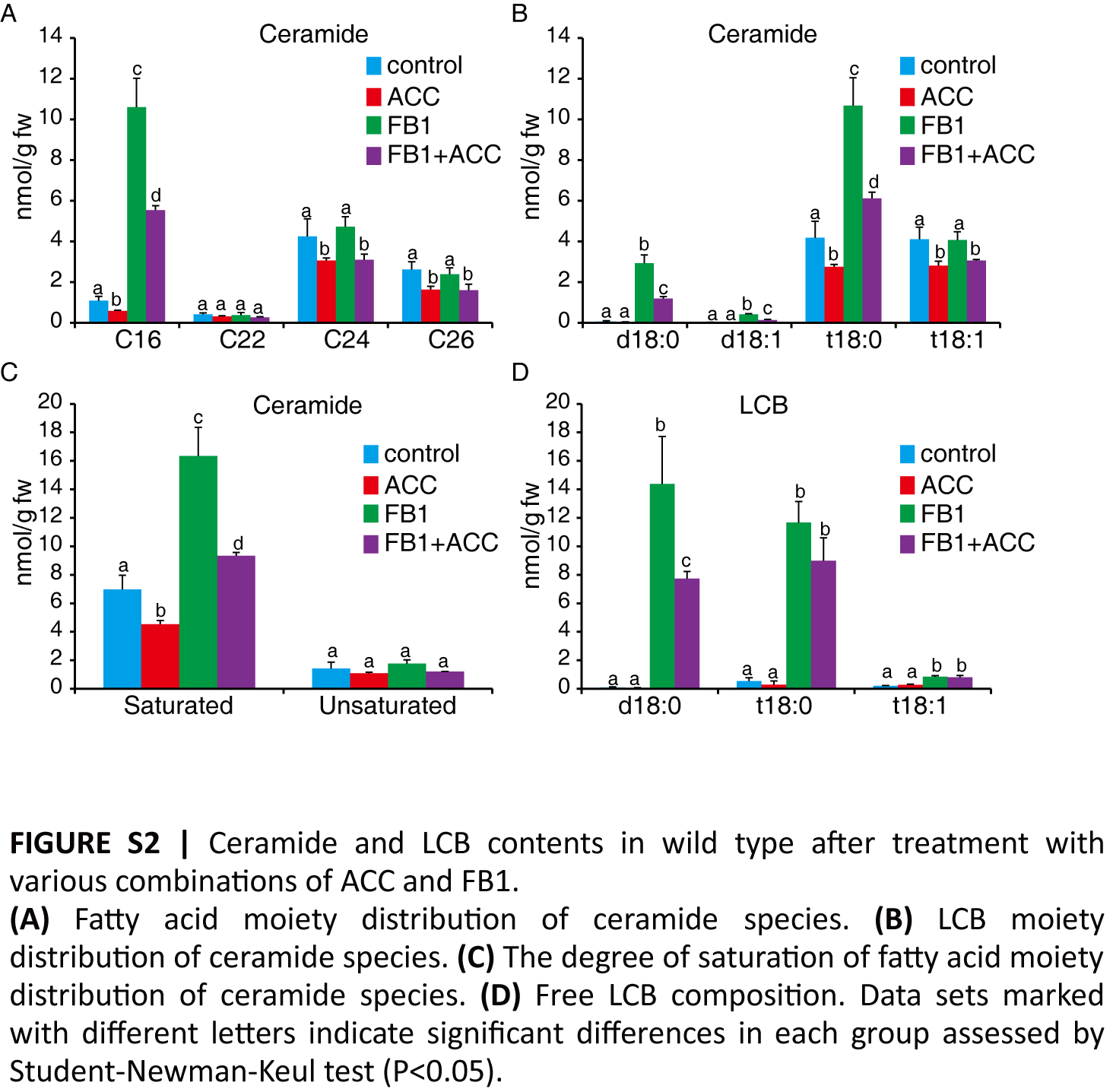

Supplement: Supplementary file 3 [file Image_2.TIF]
